# IL-22 promotes liver regeneration after portal vein ligation

**DOI:** 10.1016/j.heliyon.2024.e27578

**Published:** 2024-03-08

**Authors:** Tao Zhang, Philipp Seeger, Yashin Simsek, Morsal Sabihi, Jöran Lücke, Dimitra E. Zazara, Ahmad Mustafa Shiri, Jan Kempski, Tom Blankenburg, Lilan Zhao, Ioannis Belios, Andres Machicote, Baris Mercanoglu, Mohammad Fard-Aghaie, Sara Notz, Panagis M. Lykoudis, Marius Kemper, Tarik Ghadban, Oliver Mann, Thilo Hackert, Jakob R. Izbicki, Thomas Renné, Samuel Huber, Anastasios D. Giannou, Jun Li

**Affiliations:** a Section of Molecular Immunology and Gastroenterology, I. Department of Medicine, University Medical Center Hamburg-Eppendorf, Hamburg 20246, Germany; b Hamburg Center for Translational Immunology (HCTI), University Medical Center Hamburg-Eppendorf, 20246 Hamburg, Germany; c Department of General, Visceral and Thoracic Surgery, University Medical Center Hamburg-Eppendorf, Hamburg 20246, Germany; d Institute of Clinical Chemistry and Laboratory Medicine, University Medical Center Hamburg-Eppendorf, 20246 Hamburg, Germany; e Division for Experimental Feto-Maternal Medicine, Department of Obstetrics and Fetal Medicine, University Medical Center Hamburg-Eppendorf, Hamburg 20246, Germany; f University Children's Hospital, University Medical Center Hamburg-Eppendorf, Hamburg 20246, Germany; g Mildred Scheel Cancer Career Center HaTriCS4, University Medical Center Hamburg-Eppendorf, 20246 Hamburg, Germany; h 3rd Department of Surgery, National & Kapodistrian University of Athens, Greece; i Division of Surgery & Interventional Science, University College London (UCL), UK

## Abstract

**Background:**

Insufficient remnant liver volume (RLV) after the resection of hepatic malignancy could lead to liver failure and mortality. Portal vein ligation (PVL) prior to hepatectomy is subsequently introduced to increase the remnant liver volume and improve the outcome of hepatic malignancy. IL-22 has previously been reported to promote liver regeneration, while facilitating tumor development in the liver via Steap4 upregulation. Here we performed PVL in mouse models to study the role of IL-22 in liver regeneration post-PVL.

**Methods:**

Liver weight and volume was measured via magnetic resonance imaging (MRI). Immunohistochemistry for Ki67 and hepatocyte growth factor (HGF) was performed. IL-22 was analyzed by flow cytometry and quantitative polymerase chain reaction (qPCR) was used for acquisition of *Il-33, Steap4, Fga, Fgb* and *Cebpd*. To analyze signaling pathways, mice with deletion of STAT3 and a neutralizing antibody for IL-22 were used.

**Results:**

The remnant liver weight and volume increased over time after PVL. Additionally, we found that liver regenerative molecules, including Ki67 and HGF, were significantly increased in remnant liver at day 3 post-PVL, as well as IL-22. Administration of IL-22 neutralizing antibody could reduce Ki67 expression after PVL. The upregulation of IL-22 after PVL was mainly derived from innate cells. IL-22 blockade resulted in lower levels of IL-33 and Steap4 in the remnant liver, which was also the case in mice with deletion of STAT3, the main downstream signaling molecule of IL-22, in hepatocytes.

**Conclusion:**

IL-22 promotes liver regeneration after PVL. Thus, a combination of IL-22 supplementation and Steap4 blockade could potentially be applied as a novel therapeutic approach to boost liver regeneration without facilitating tumor progression after PVL.

## 1 Introduction

Malignant lesions of the liver include primary tumors, for example, hepatocellular carcinoma (HCC) and intrahepatic cholangiocarcinoma (iCCA), as well as liver metastasis originating from other sites, most commonly from colorectal cancer (CRC) [1]. Among current therapeutic options, complete surgical resection of the lesions provides a curative approach [[2], [3], [4]]. However, insufficient future liver remnant volume (FLR) after liver segment resection leads to postoperative liver failure, a severe complication associated with increased mortality rates [[5], [6], [7]]. To boost liver regeneration, portal vein ligation (PVL) and embolization (PVE) have been utilized to increase FLR. By ligating or embolizing the branches of the portal vein that supply the cancer-affected section of the liver, the remaining tissue proliferates, gaining on average around 40 % of its previous volume [8,9]. This method has been shown to improve outcomes in patients with colorectal cancer-derived liver metastasis by increasing the chance to perform a curative surgical resection of liver metastasis [10].

Comparisons focusing on resection of colorectal cancer liver metastasis (CRLM) with and without prior PVE/PVL, higher recurrence rates, and accelerated tumor growth after induction of liver regeneration have been a topic of discussion. Several studies report a lower disease-free survival rate after first stage PVE, although overall survival does not seem to be affected in this case [11,12]. In a different perspective study with adjusted analysis for patient characteristics, no difference in DFS or OS were reported [13], confirmed by a systematic review [10]. Despite this, one of the main reasons for the failure of second-stage resection is disease progression (both intrahepatic and extrahepatic) [11,[14], [15], [16]]. Furthermore, CRLM and HCC are reported to increase in malignant volume after PVE [12,[16], [17], [18], [19]], although this could not be confirmed in an animal model [20]. Further detailed investigations are required to elucidate the possible connection between tumor progression and PVL/PVE, the mechanisms of liver regeneration and their effect on cancer growth. Interestingly, several molecular pathways mediating liver regeneration overlap with the promotion of CRLM recurrence [[21], [22], [23]]. In this paper, we aim to integrate our previous findings regarding IL-22-mediated hepatocyte proliferation and HCC progression to further describe an approach that could be utilized to balance simultaneous liver regeneration and suppressed tumor progression.

IL-22, a cytokine from the IL-10 family, is mainly produced by Th17, Th22, iNKT cells, and ILCs [24] and has been demonstrated to play a role in hepatocyte proliferation [25]. By binding to its receptor complex (IL-22Ra1) on hepatocytes, IL-22 induces the expression of mitogenic and antiapoptotic proteins, via the activation of the STAT3 signaling pathway [[25], [26], [27]]. Blocking IL-22 leads to a decrease in hepatocyte proliferation after hepatectomy in mice [25]. While exerting protective effects in acute liver injury, IL-22 is reported to have a pro-tumorigenic effect in HCC [25,28].

In our recent publication regarding the role of IL-22 in HCC progression, we found five genes that were upregulated after IL-22 stimulation upon murine hepatocytes *in vitro*, namely *Steap4, Il-33, Fga, Fgb,* and *Cebpd* [29]. Furthermore, we identified a positive correlation between *Steap4* expression and IL-22 levels in HCC tissue *in vivo*, thus identifying a possible downstream mechanism regarding IL-22 and hepatocyte proliferation. This connection has so far not been investigated in the PVL procedure.

Here, we adapted a murine model for PVL described by Schlegel et al. [30] to investigate how IL-22 acts on liver regeneration after PVL. We found the model to be viable as a significant increase in future liver remnant volume was seen via imaging, and an increase in hepatocyte proliferation was identified by Ki67 and hepatocyte growth factor (HGF) measurements. Furthermore, we identified that IL-22 was majorly upregulated in innate cells after PVL. Of note, IL-22 blockade significantly reduced Ki67 expression, a marker for proliferation, in remnant liver. Mechanistically, IL-22 blockade and impaired STAT3 signaling in hepatocytes resulted in lower levels of *Il33* and *Steap4* in the remnant liver. Thus, we describe a new pathway for hepatocyte proliferation after PVL. Therefore, blocking Steap4 in addition to supplementation with IL-22 could be a potential treatment regime to boost liver regeneration without triggering tumor progression after PVL.

## 2 Materials and methods

### 2.1 Animals

*C5*7BL*/6*, *Foxp*^*mRFP*^*; Il10*^*eGFP*^*;Il22*^*BFP*^*; Il17*^*Katushka*^*; CD45.1/2*; *Il17a*^*eGFP*^*; Il17f*^*mRFP*^*, Stat3*^*flox/flox*^*;Alb*^*Cre*^^+^ *mice* were bred and housed under specific pathogen-free conditions in the animal facility of the University Medical Center Hamburg- Eppendorf. Age- and sex-matched littermates between 8 and 16 weeks of age were used. Animal experiments were carried out in accordance with the Institutional Review Board “Behörde für Soziales, Familie, Gesundheit und Verbraucherschutz” (Hamburg, Germany).

### 2.2 Surgical procedure

Mice were anesthetized using isoflurane inhalation and received buprenorphine (100 μg/kg) subcutaneously (s.c.) for analgesia. After a midline laparotomy, the right posterior, left lateral, left medial, and caudal portal vein branch was ligated using 9-0 silk sutures under a microscope, which corresponds to a 30% future liver remnant volume (FLR) (or 70% ligated liver volume) [31]. Successful ligation was confirmed by visual control of parenchyma discoloration [30]. The abdominal wall and skin were sutured separately. After the operation, we maintained analgesia by daily s.c. injection of buprenorphine (100 μg/kg) as well as application of metamizole (240 mg/250 ml) within the drinking water for 3 days. IL-22 neutralizing antibody (50ug/mouse, R&D Systems) was administrated 1 day intraperitoneally post-PVL [32].

### 2.3 MRI imaging

Hepatic volumes were measured by using a 7 T MRI scanner (ClinScan; Bruker BioSpin, Ettlingen, Germany) to assess liver regeneration after PVL. During the procedure, mice were under anesthesia using isoflurane inhalation. Liver volume was further assessed by the image processing program ImageJ (U.S. National Institutes of Health).

### 2.4 Analysis of liver transaminases and white blood cell count

Blood samples were collected by punction of the vena cava during liver harvest (see next paragraph) and ALT, AST, and ALP enzyme levels as well as neutrophile, monocyte, and platelet count were analyzed at the Department of Clinical Chemistry (University Medical Center Hamburg- Eppendorf).

### 2.5 Quantification of plasma DNA

Plasma DNA was quantified based on the described method. 6 μL of plasma was diluted in 114 μL of PBS containing 0.1% bovine serum albumin. 50 μL of diluted plasma was then mixed with 50 μL PBS containing Sytox™ Green nucleic acid stain (Invitrogen) at a final concentration of 2 μM to label DNA fluorescently. Fluorescence was recorded using MTP reader (Tecan Genios) with a 485 nm excitation and 535 nm emission filter set. Autofluorescence was considered as background and determined in samples mixed with PBS without Sytox™ Green. DNA concentrations were calculated based on a standard curve obtained by known concentrations of DNA (Thermo Scientific).

### 2.6 Immune cell isolation

Mice were sacrificed at the same time point and the livers were perfused with ice-cold PBS through the portal vein and the vena cava. The gall bladder was excised completely. For immune cell isolation, the liver was cut into small pieces and digested in 6 ml RPMI 1640 (Thermo Fisher Scientific, Waltham, MA, USA) supplemented with 100 U/ml collagenase, 10% FBS, 1 mM CaCl_2_, 1 mM MgCl_2_, and incubated while shaking for 25 min at 37 °C. The liver suspension was then passed through a 100 μm metal strainer and washed with PBS/1% FBS (Thermo Fisher Scientific). After centrifugation at 400 g and 4 °C for 8 min, the pellet was resuspended in 4 ml of 40% Percoll (Sigma-Aldrich, Darmstadt, Germany) and slowly transferred on top of 4 ml 67% Percoll, followed by centrifugation for 25min at 400 g and 4 °C with slow acceleration and deceleration. The interphase was washed once in 50 ml PBS/1% FBS and ready for further staining.

### 2.7 Cell staining and flow cytometry

Isolated immune cells were stained with fluorochrome-conjugated antibodies (Table 1) for extracellular markers for 15 min. After washing, stained cells were analyzed by using a BD LSRFortessa Cytometer (BD, Franklin Lakes, NJ, USA). Data were analyzed using FlowJo 9 (FlowJo LLC, Ashland, OR, USA).

**Table 1:** Antibodies used for cell staining in flow cytometry.

Target	Manufacturer	Catalog number
CD45	Biolegend	103,138
CD3	Biolegend	100,206
CD4	BD	612,761
CD8	Biolegend	100,722
Live/dead	Biolegend	423,107

### 2.8 RNA isolation and real-time PCR

Total RNA isolation from the liver was performed using Trizol reagent (Applied Biosystems, Waltham, MA, USA) according to manufacturer instructions. cDNA synthesis was carried out with a high-capacity cDNA reverse transcription kit (Applied Biosystems). Real-time PCR was performed using a StepOnePlus Real-Time PCR System (Applied Biosystems). The probes (TaqMan, Applied Biosystems) were *Hprt* Mm03024075_m1, *Il33* Mm03024075_m1, *Steap4* Mm00475405_m1, *Fga* Mm00802584_m1, *Fgb* Mm00805336_m1, *Cebpd* Mm00786711_s1.

### 2.9 H&E staining

Liver specimens were immediately fixed in 4% buffered formalin after excision, and later embedded in paraffin or OCT (Sakura, Tokyo, Japan) and stored at −80 °C. Tissue sections (4 mm) were prepared and stained with H&E for microscopic pictures. Necrotic areas and neutrophils were quantified by area fraction analysis software (ImageJ, U.S. National Institutes of Health, Bethesda, MD, USA).

### 2.10 Immunohistochemical staining and analysis

Formalin-fixed and paraffin-embedded liver sections were deparaffinized in xylene and graded alcohol solutions, hydrated, and then washed in PBS. Sections were heat-treated with 10 mM sodium citrate buffer (pH = 6), then with 3% H_2_O_2_ for 20 min to inhibit endogenous peroxidase. Incubation was done with a protein-blocking agent in PBS with 5% FCS and 10% BSA. Tissue slides were then stained with monoclonal Ki-67 antibodies and neutrophile antibodies (ab21595, Abcam, Cambridge, UK). The Envision Detection System-HRP (Dako, Agilent, Santa Clara, CA, USA) was used to identify the primary antibody. High-resolution images (HRI) of the whole liver section were obtained with a high-resolution slide scanner (NanoZoomer 2.0-HT Digital slide scanner). Protein expression was further assessed by the image processing program ImageJ (U.S. National Institutes of Health). HRI or extracted parts were quantified by taking the average value of manually counted stained cells in 10 randomly selected Fields of View (FOV) per specimen by a blinded independent assistant.

### 2.11 Statistical analysis

Statistical analysis was performed using GraphPad Prism Software (GraphPad Software, San Diego, CA, USA). For the comparison of groups, the nonparametric two-sided Mann–Whitney or Student t-test was used. Bonferroni correction was used to adjust the p-value in case of multiple comparisons. For time-dependent liver regeneration data, a repeated-measures ANOVA to assess the significance of the main effects and an experimental group-time interaction was used. Pearson correlation was used for correlative analyses. The significance level for all analyses was set to p < 0.05.

## 3 Results

### 3.1 Proliferation molecules increased and peaked at day 3 in the remnant liver after PVL

**Fig. 1:**
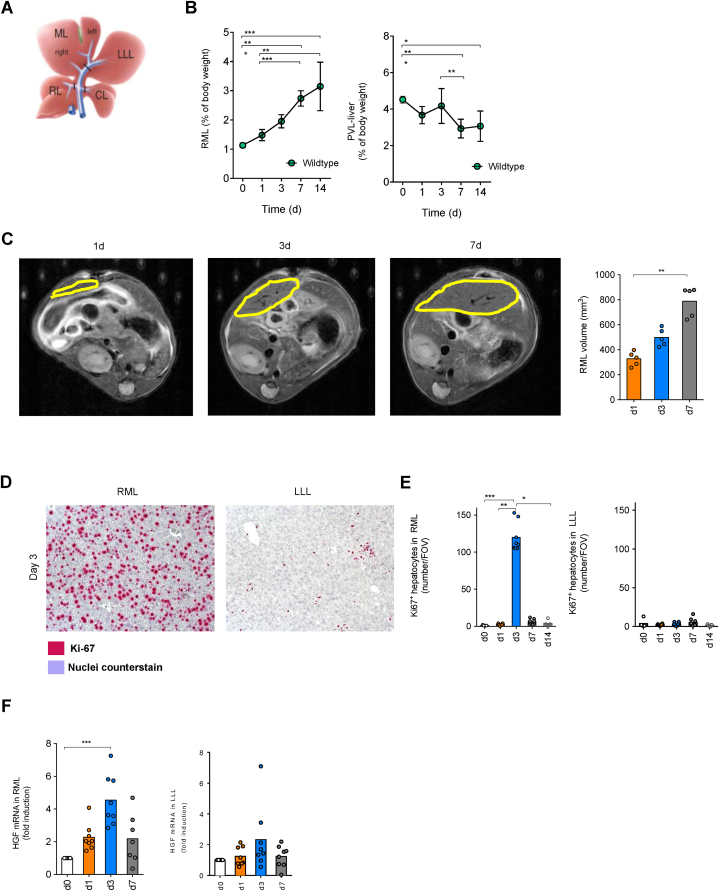
Proliferation molecules increased and peaked at day 3 in the remnant liver after PVL. (**A**) Schematic view of PVL. (**B**) Weight of right medial lobe (left) and ligated lobes (right) relative to body weight over 14 days after PVL (d0, n = 5; d1, n = 8; d3, n = 8; d7, n = 13, d14, n = 5). (**C**) MR-imaging on wildtype mice underwent PVL. The volume of the remnant liver (RML) was quantified (n = 5). Representative picture of immunohistochemistry staining (**D**) and number of Ki-67-positive hepatocytes (**E**) per field of view of RML and LLL over 14 days after PVL (n = 8). Scale bar 100 μm. (**F**) Fold induction of hepatocyte growth factor (HGF) mRNA normalized to Hprt in RML and LLL after PVL. (n = 8). Data presented as mean ± SEM. ns: p > 0.05; *: p < 0.05; **: p ≤ 0.01; ***: p ≤ 0.001 as assessed by One-way ANOVA and Bonferroni correction. RL, right lobe; ML, medial lobe; LLL, left lateral lobe; CL, caudate lobe; RML, right medial lobe; FLR, future remnant liver volume; PVL, portal vein ligation; FOV, the field of view; HGF, hepatocyte growth factor.

We performed PVL in wildtype mice and monitored liver weight changes in both remnant liver and ligated liver (Fig. 1A). We observed a continuous increase of RML weight normalized by body weight for up to 14 days, while the normalized weight of ligated liver decreased over time (Fig. 1B). Besides this, we examined the RML volume using MR imaging and found the remnant liver to become enlarged over time (Fig. 1C). To investigate if this volume and weight increase in RML was due to the proliferation of liver cells instead of hypertrophy, we performed immunohistochemistry on liver sections. We observed a high expression of Ki-67 in the remnant liver, as well as almost undetectable Ki-67+ cells in the ligated liver at day 3 post-PVL (Fig. 1D). Furthermore, we analyzed the number of Ki-67+ hepatocytes in the liver section at different time points. Surprisingly, the most enriched Ki-67+ hepatocytes in the remnant liver were present at day 3 post-PVL, while Ki-67 expression remained at a low level in the ligated liver (Fig. 1E). Of note, proliferation molecule HGF also increased in the remnant liver and peaked at day 3 post-PVL (Fig. 1F). By using this mouse model for PVL, we found that the remnant liver regenerated over time, and proliferation molecules reached the highest level at day 3 post-PVL.

### 3.2 Liver damage after PVL resulted in drastic liver transaminases release on day 1

**Fig. 2:**
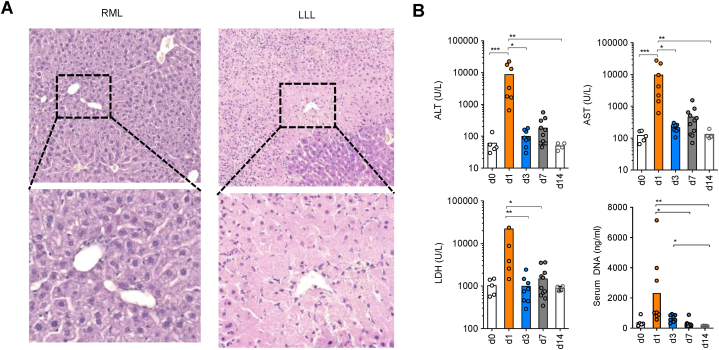
Liver damage after PVL resulted in drastic liver transaminases release on day 1 (**A**) Representative images of HE-stained sections of RML and LLL liver tissue 1 day after PVL. Scale bar 100 μm. (**B**) Alanine transaminase (ALT, top left), aspartate aminase (AST, top right), lactate dehydrogenase (LDH, bottom left) activity in serum measured in U/L and DNA-levels in serum (bottom right) measured in ng/ml over a time course of 14 days (d0, n = 5; d1, n = 7; d3, n = 8; d7, n = 11; d14, n = 4). Data presented as mean ± SEM. ns: p > 0.05; *: p < 0.05; **: p ≤ 0.01; ***: p ≤ 0.001 as assessed by One-way ANOVA and Bonferroni correction. HE, hematoxylin and eosin stain; ALT, alanine transaminase; AST, aspartate aminase; LDH, lactate dehydrogenase.

PVL not only induced liver regeneration to the remnant liver but also led to necrosis in the ligated liver due to a lack of blood supply (Fig. 2A). To identify whether this damage and the subsequent effect was continuous, we collected serum from mice under PVL from different time points and measured the levels of liver transaminases. Interestingly, all liver transaminases including ALT, AST, and LDH increased drastically 1 day after PVL, and dropped to comparable levels seen in unoperated mice from day 3 and onwards (Fig. 2B). Additionally, the peak level of cell-free DNA in murine serum was also observed at day 1 post PVL (Fig. 2B). Taken together, liver transaminases and cell-free DNA release caused by PVL peaked at day 1 post-PVL and dropped back to normal levels as in unoperated mice shortly after.

### 3.3 Neutrophils were enriched in the ligated liver after PVL

**Fig. 3:**
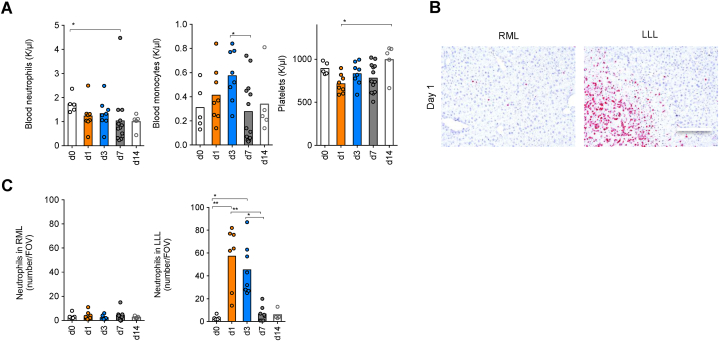
Neutrophils were enriched in the ligated liver after PVL. (**A**) Peripheral blood levels of neutrophils, monocytes, and platelets at days 0, 1, 3, 7, and 14 after PVL (d0, n = 5; d1, n = 8; d3, n = 8; d7, n = 13; d14, n = 5*)*. Immunohistochemistry (scale bar 100 μm) (**B**) and number (C) of neutrophils per field of view of RML and LLL liver tissue on day 0 (n = 5), 1 (n = 7), 3 (n = 8), 7 (n = 8) and 14 (n = 4) after PVL. Data presented as mean ± SEM. ns: p > 0.05; *: p < 0.05; **: p ≤ 0.01; ***: p ≤ 0.001 as assessed by One-way ANOVA and Bonferroni correction.

White blood cells have been reported to be involved with liver diseases and liver regeneration [33,34]. To this end, we also collected blood from wildtype mice that underwent PVL and counted white blood cells. Blood neutrophils and platelet counts were not altered after PVL. Despite a slight increase in monocyte count after PVL, this value did not reach significance (Fig. 3A). Since neutrophils were identified as an important element contributing to liver regeneration, especially after partial hepatectomy [[35], [36], [37]], we further performed staining of neutrophils in the liver section. Surprisingly, neutrophils were not enriched in the remnant liver up to 14 days after PVL. However, neutrophil infiltration was observed in the ligated liver since day 1 post-PVL and gradually decreased over time (Fig. 3B and C). Taken together, white blood cells were not changed after PVL, while neutrophils were enriched exclusively in the ligated liver.

### 3.4 An increase of IL-22 in innate cells was found in the remnant liver at day 3 post-PVL

**Fig. 4:**
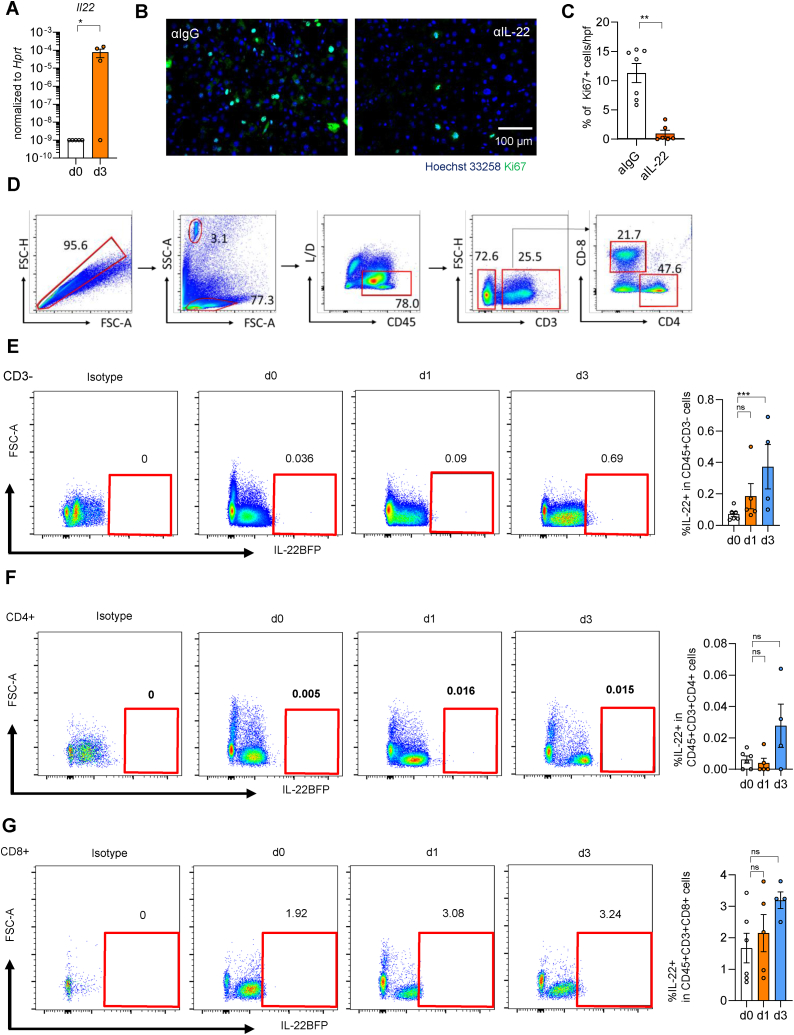
An increase of IL-22 in innate cells was found in the remnant liver at day 3 post-PVL. (**A**) mRNA level of Il-22 in remnant liver at day 3 post-PVL (n = 4–5). Representative picture of Ki-67-immunohistochemistry staining **(B)** and number of Ki-67-positive hepatocytes **(C)** per field of view of RML at 3 days post-PVL after injection of anti-IL-22 antibody (n ≥ 6). (**D-F**) Flow cytometry analysis of immune cells isolated from RML liver tissue at day 0 (n = 6), 1 (n = 5), and 3 (n = 4) after PVL using IL-22 BFP reporter mice. (**D**) Gating strategy for the isolation of CD3^−^cells as well as CD4^+^ and CD8^+^ T-cells, representative data sets. Representative FACS plot for frequency of IL-22+ cells in CD3^−^cells (**E**), CD4^+^ cells (**F**), and CD8^+^ cells (**G**) at indicated time points. Data presented as mean ± SEM. ns: p > 0.05; *: p < 0.05; **: p ≤ 0.01; ***: p ≤ 0.001 as assessed by One-way ANOVA and Bonferroni correction.

Previously we reported that IL-22 could promote the proliferation of hepatocytes [29]. And indeed, we observed upregulation of IL-22 in the remnant liver at day 3 post-PVL (Fig. 4A). Functionally, neutralizing IL-22 starting from day 1 pre-PVL could significantly reduce the Ki-67+ cells in the remnant liver (Fig. 4B and C). Here, we performed PVL using reporter mice and analyzed IL-22 levels among distinct cell populations. The majority of immune cells here were classified as innate cells (Fig. 4D). In addition, IL-22 expression in innate cells increased significantly at day 3 post-PVL, compared to pre-surgery (Fig. 4E). Even though we observed an upregulation of IL-22 expression in CD4^+^ and CD8^+^ T cells throughout the procedure, these values did not reach significance at day 3 post-PVL (Fig. 4F and G). Taken together, IL-22 promoted liver regeneration, and the cellular source of IL-22 upregulation in the remnant liver at day 3 post-PVL is described as innate cells.

### 3.5 IL-22 neutralizing and ablation of STAT3 signaling in hepatocytes downregulated *Il33* and *Steap4*

**Fig. 5:**
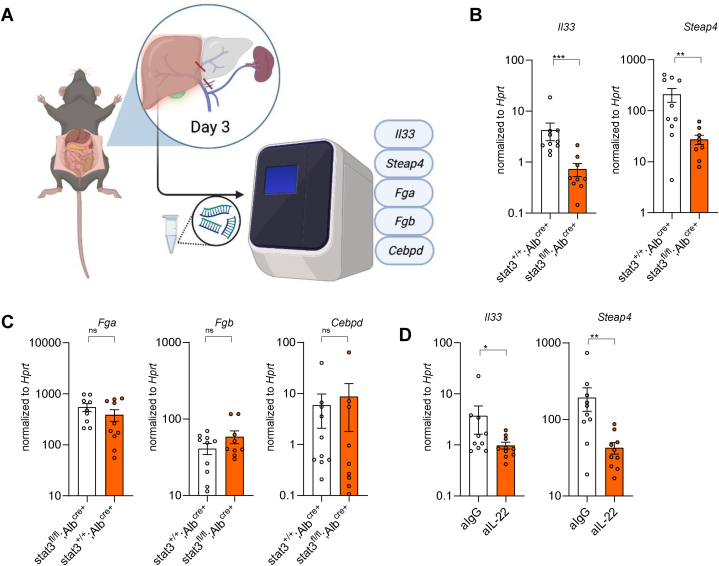
IL-22 neutralizing and ablation of STAT3 signaling in hepatocytes downregulated *Il-33* and *Steap4*. (**A**) Schematic view of PVL in *Stat3*^*flox/flox*^*;Alb*^*Cre+*^ and *Stat3*^*wt/wt*^*;Alb*^*Cre*^^*+*^ mice. (**B, C**) Analysis of qPCR in RML of *Stat3*^*flox/flox*^*;Alb*^*Cre+*^ and *Stat3*^*wt/wt*^*;Alb*^*Cre*^^*+*^ mice after PVL (n ≥ 9). (**D**) Analysis of qPCR in RML of mice receiving IL-22 neutralizing antibody (n ≥ 9). Data presented as mean ± SEM. ns: p > 0.05; *: p < 0.05; **: p ≤ 0.01; ***: p ≤ 0.001 as assessed by Mann–Whitney *U* test.

Previously, we showed that IL-22 can promote the proliferation of hepatocytes and analyzed gene expression profiles in murine hepatocytes after IL-22 stimulation *in vitro* [29]. Several candidates that were upregulated by IL-22 stimulation were further validated using qPCR, including *Il33*, *Steap4, Fga, Fgb, and Cebpd* [29]. Many studies have shown that activation of IL-22 signaling is induced through the phosphorylation of STAT3 in target cells [[25], [26], [27],[38], [39], [40], [41]]. Thus, we examined these candidates in mice with STAT3 ablation in hepatocytes and performed qPCR (Fig. 5A). Strikingly, we saw a reduction of *Il33* and *Steap4* when STAT3 signaling was impaired in hepatocytes, while expression of *Fga, Fgb, and Cebpd* was not altered (Fig. 5B–C). Moreover, we found that *Il33* and *Steap4 were indeed reduced in mice* receiving an IL-22-neutralizing antibody (Fig. 5D). Collectively, these results show that IL-22 neutralizing and STAT3 deficiency in hepatocytes downregulated IL-33 and Steap4 in the remnant liver after PVL.

## 4 Discussion

In this study, we investigated the mechanism underlying IL-22-mediated liver regeneration after PVL. Using a mouse model, we determined the peak time of liver regeneration molecules in the remnant liver, as well as of liver damage molecules released into the circulation system. Moreover, we identified the cellular source of IL-22 in the remnant liver after PVL. Furthermore, we suggested that IL-22 promoted hepatocyte proliferation via *Il-33* and *Steap4* upregulation in the context of PVL.

We observed a dynamic increase in both the volume and weight of the murine remnant liver after PVL for up to 14 days. Interestingly, the peak time of Ki-67 and HGF in the remnant liver was both at day 3 post-PVL, suggesting an important time window for responsible modulators during liver regeneration. In addition, the liver damage molecules were mostly released one day after PVL, and became drastically diminished to the normal level as unoperated. This provides insight into when clinical treatment against liver failure may be most efficient for patients shortly after undergoing PVL surgery.

Among distinct immune cell types, the role of white blood cells has been reported to be significant in liver regeneration [[42], [43], [44]]. However, we did not find a significant change in white blood cells in the circulation of mice after PVL. Neutrophils, as the major population in white blood cells, were reported to contribute to liver regeneration after partial hepatectomy via interaction with intracellular adhesion molecules (ICAM-1) [43]. Thus, we further examine the infiltrated neutrophils in the remnant and ligated liver sections. Surprisingly, neutrophils were more infiltrated in the ligated liver while almost undetectable in the remnant liver for up to 14 days. This neutrophil recruitment in the ligated liver could be due to the remaining blood supply from the hepatic artery and vein, which was also observed during liver ischemia-reperfusion [45]. Overall, white blood cells do not seem to modulate liver regeneration after PVL.

We previously showed that IL-22 could promote hepatocyte proliferation [29]. To examine if IL-22 was upregulated in the remnant liver after PVL, we performed qPCR and found that *Il22* was upregulated in the remnant liver. To further investigate the cellular source of IL-22 upregulation, we performed flow cytometry. We looked into lymphocytes, the major source of IL-22, and found out that IL-22 was indeed increased in innate cells at day 3 post-PVL, consistent with the peak time of Ki-67 and HGF. Strikingly, blocking IL-22 in mice after PVL reduced the expression of liver regenerative molecule Ki-67 in the remnant liver. This enhanced the role of IL-22 in liver regeneration after PVL. However, IL-22 not only presented as a driver for liver regeneration, but also a tumorigenic modulator in liver malignancy [29]. To set the basis for a future therapy making the best use of IL-22 on liver regeneration without triggering tumor progression, we analyzed the gene expression changes in the remnant liver upon IL-22 neutralizing post-PVL. Strikingly, we found that blocking IL-22 downregulated *Il33* and *Steap4* in the remnant liver after PVL. Of note, this is consistent with findings from mice with impaired STAT3 signaling in hepatocytes. Nevertheless, we previously reported IL-22 could increase *Steap4* in hepatocytes, leading to malignancy [29]. So far, IL-33 signaling was more studied in the respiratory system and found to modulate IL-22-dependent antibacterial defense during respiratory infection [46,47]. We studied the relation of IL-22 and IL-33 in the liver, and found that blocking IL-22 downregulated Il33 in the remnant liver after PVL. Taken together, IL-22-regulated *Il33* was specifically related to hepatocyte proliferation, whereas *Steap4* was involved both in hepatocyte proliferation and tumor progression.

*Steap4* was reported as a potential marker for immune infiltration in colorectal cancer [48]. However, the role of Steap4 has not been tested *in vivo*. More studies involving models that combine tumor progression and PVL have to be conducted to prove the effect of this concept.

Zhang et al. [49] showed that depletion of Kupffer cells attenuates the parenchyma growth after a special ALPPS procedure in mice, accompanied by lower tumor necrosis factor alpha (TNF-a) and interleukin 6 (IL-6) levels. Additionally, the balance of Gata3 and Ramp2 was shown to influence liver regeneration after hepatectomy in mice via enhancing secretion of vascular endothelial growth factor (VEGF) to induce vascularization [50]. The involvement of VEGF was also seen by Zhao et al. [51]. According to these results, liver hypertrophy after PVL or other surgical alterations works via multiple pathways. Further research is warranted to test these substances in clinical practice to support liver regeneration without promoting tumor growth.

In conclusion, this study revealed that innate cell-derived IL-22 can promote hepatocyte proliferation after PVL. Moreover, a combination of IL-22 supplementation and Steap4 blockade could possibly serve as a novel therapeutic approach to boost liver regeneration without facilitating tumor progression after PVL.

## Limitations

To analyze the effect of IL-22 after PVL on tumor progression, a model for hepatic malignancy should be used, for example the injection of cancer cells in the portal vein. The effect of in situ transection of the liver, as it is used in humans in context of the ALPPS procedure (“Associating Liver Partition and Portal Vein Ligation for Staged Hepatectomy”), was not examined in this study.

## Ethics statement

Animal experiments were carried out in accordance with the Institutional Review Board and ethics comittee “Behörde für Soziales, Familie, Gesundheit und Verbraucherschutz” (reference numbers for approval G53/2017 and N33/2021) and conducted in accordance with institutional guidelines of animal husbandry and welfare.

## Funding

This research was funded in part by the 10.13039/501100001659Deutsche Forschungsgemeinschaft (grant SFB841 to S.H.), Else Kröner Memorial Stipendium (to A.D.G), the 10.13039/501100004038Jung Foundation for Science and Research (Ernst Jung Career Development Award 2022) (to A.D.G), China Council Scholarship (to T.Z.). S.H. has an endowed Heisenberg Professorship awarded by the 10.13039/501100001659Deutsche Forschungsgemeinschaft.

## Informed consent statement

Informed consent was obtained from all subjects involved in the study.

## Data availability statement

Data are included within the article or its Supplementary Materials. RNA sequencing data will be provided upon reasonable request.

## CRediT authorship contribution statement

**Tao Zhang:** Writing – review & editing, Writing – original draft, Visualization, Validation, Software, Resources, Methodology, Investigation, Formal analysis, Conceptualization. **Philipp Seeger:** Writing – review & editing, Writing – original draft, Software, Methodology, Formal analysis, Data curation, Conceptualization. **Yashin Simsek:** Writing – original draft, Software, Methodology, Formal analysis, Conceptualization. **Morsal Sabihi:** Writing – review & editing, Validation, Methodology. **Jöran Lücke:** Validation, Resources, Methodology, Investigation. **Dimitra E. Zazara:** Writing – review & editing, Validation, Methodology. **Ahmad Mustafa Shiri:** Validation. **Jan Kempski:** Validation, Methodology. **Tom Blankenburg:** Validation, Methodology, Formal analysis. **Lilan Zhao:** Validation, Methodology. **Ioannis Belios:** Validation, Methodology. **Andres Machicote:** Validation, Methodology. **Baris Mercanoglu:** Validation, Methodology. **Mohammad Fard-Aghaie:** Validation, Methodology. **Sara Notz:** Validation, Methodology. **Panagis M. Lykoudis:** Validation, Methodology. **Marius Kemper:** Validation, Methodology. **Tarik Ghadban:** Validation, Methodology. **Oliver Mann:** Writing – review & editing. **Thilo Hackert:** Writing – review & editing. **Jakob R. Izbicki:** Writing – review & editing. **Thomas Renné:** Writing – review & editing, Methodology. **Samuel Huber:** Resources, Investigation. **Anastasios D. Giannou:** Writing – review & editing, Writing – original draft, Visualization, Validation , Supervision, Software, Resources, Project administration, Methodology, Investigation, Funding acquisition, Formal analysis, Data curation, Conceptualization. **Jun Li:** Supervision.

## Declaration of competing interest

The authors declare that they have no known competing financial interests or personal relationships that could have appeared to influence the work reported in this paper.
