# Supplementary material for: IL-22 promotes liver regeneration after portal vein ligation
Source: Heliyon. 2024 Mar 8;10(6):e27578. doi: 10.1016/j.heliyon.2024.e27578 (PMC10963228; doi:10.1016/j.heliyon.2024.e27578)
Supplement: Multimedia component 1 [file mmc1.docx]

**IL-22 promotes liver regeneration after portal vein ligation**

Tao Zhang MD^1,2,#^, Philipp Seeger MD ^3,#^, Yashin Simsek MD^4,#^, Morsal Sabihi^1,2^, Jöran Lücke MD^1,2,3^, Dimitra E. Zazara MD^5,6^, Ahmad Mustafa Shiri^1^, Jan Kempski MD^1,2,7^, Tom Blankenburg^1,2^, Lilan Zhao MD^3^, Ioannis Belios^5^, Andres Machicote PhD^1,2^, Baris Mercanoglu MD^3^, Mohammad Fard-Aghaie MD^3^, Sara Notz MD^3^, Panagis M. Lykoudis MD^8,9^, Marius Kemper MD^3^, Tarik Ghadban MD^3^, Oliver Mann, Professor, MD^3^, Thilo Hackert, Professor, MD^3^, Jakob R. Izbicki, Professor, MD^3^, Thomas Renné, Professor, MD^4^, Samuel Huber, Professor, MD^1,2,†^, Anastasios D. Giannou MD PhD^1,2,3,†,*^, and Jun Li, Professor, MD ^3,†,*^.

Two-Sentence Summary

In a mice model for portal vein ligation, IL-22 promotes liver regeneration via STAT3 and Steap4 signaling, which have previously shown to facilitate hepatic tumor development. This finding can be important when applied as a novel therapeutic approach to boost liver regeneration without facilitating tumor progression after PVL by a combination of IL-22 supplementation and Steap4 blockade.
